# Supplementary figures and images for: Dynamics of data availability in disease modeling: An example evaluating the trade-offs of ultra-fine-scale factors applied to human West Nile virus disease models in the Chicago area, USA
Source: PLoS One. 2021 May 19;16(5):e0251517. doi: 10.1371/journal.pone.0251517 (PMC8133451; doi:10.1371/journal.pone.0251517)

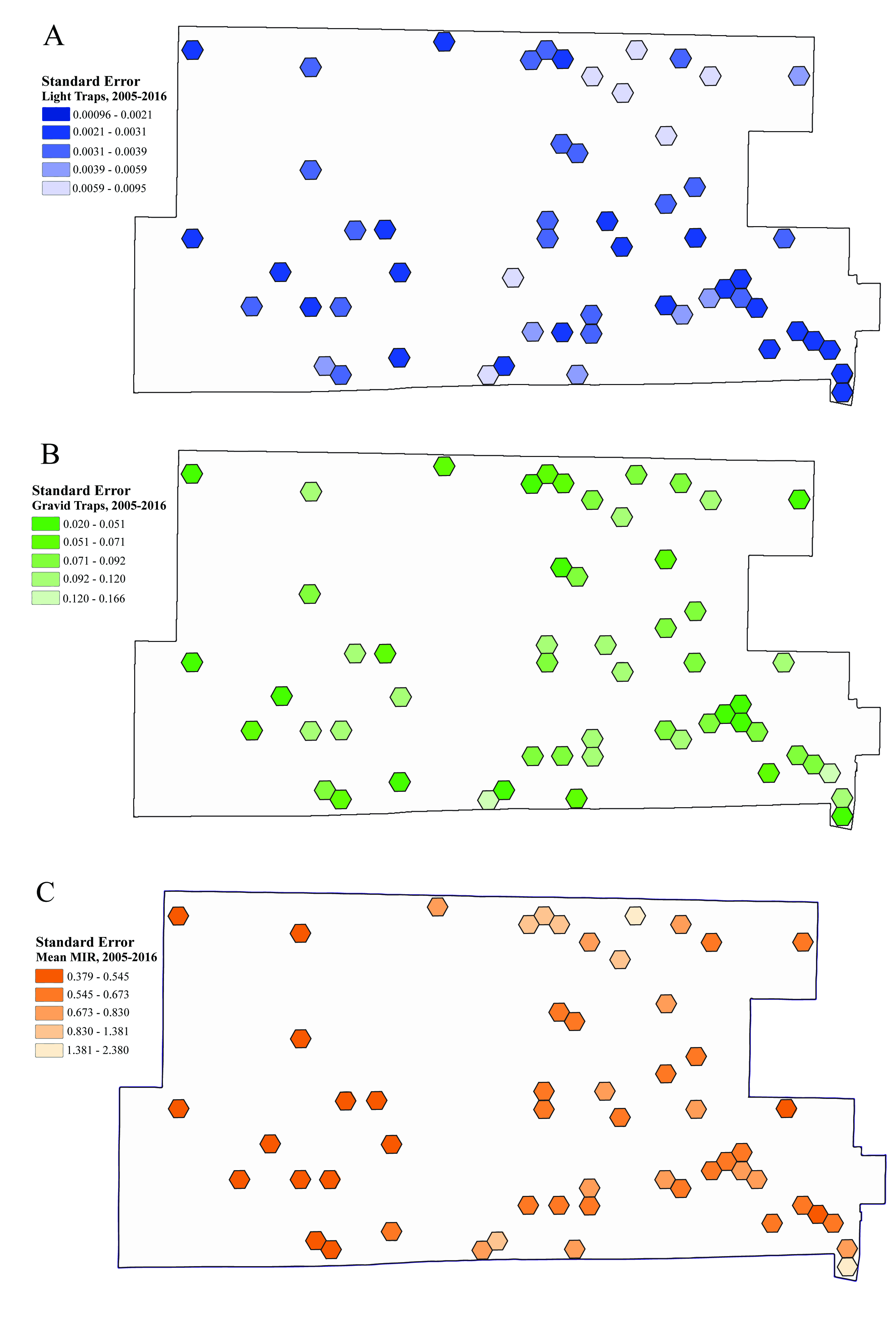

Supplement: S1 Fig — Measurement of standard error associated with interpolated Culex species abundance by light (A) and gravid (B) traps (averaged for all traps), and mean MIR (C) for each of the 55 hexagons, from 2005–2016. The average weekly mosquito abundance multiplied by average weekly mean MIR created a third infection parameter, the vector index. (TIF) [file pone.0251517.s001.tif]
