# Supplementary material for: Dynamics of data availability in disease modeling: An example evaluating the trade-offs of ultra-fine-scale factors applied to human West Nile virus disease models in the Chicago area, USA
Source: PLoS One. 2021 May 19;16(5):e0251517. doi: 10.1371/journal.pone.0251517 (PMC8133451; doi:10.1371/journal.pone.0251517)
Supplement: S2 Table — Tables are grouped by anthropogenic (A), biological (B), environmental (C), weather (D), or other (E), as indicated in Table 1. (DOCX) [file pone.0251517.s003.docx]

**A.**

**C.**

**B.**

**E.**

**D.**
