# Supplementary material for: Dynamics of data availability in disease modeling: An example evaluating the trade-offs of ultra-fine-scale factors applied to human West Nile virus disease models in the Chicago area, USA
Source: PLoS One. 2021 May 19;16(5):e0251517. doi: 10.1371/journal.pone.0251517 (PMC8133451; doi:10.1371/journal.pone.0251517)
Supplement: S1 Methods — (DOCX) [file pone.0251517.s008.docx]

**S1 Methods**

**Previously existing data**

**Human illness**

Human WNV cases in Illinois were classified as either confirmed or probable, as reported to the IDPH by public health or licensed medical professionals (mandatory reporting of WNV cases is required in the state). The case definition for a confirmed case of arboviral encephalitis in Illinois is a clinically compatible illness that is laboratory confirmed at a public health laboratory. The laboratory criteria are a fourfold or greater rise in serum antibody titer; or isolation of virus from, or demonstration of viral antigen in, tissue, blood, cerebrospinal fluid (CSF) or other body fluid; or specific IgM antibody in CSF. A probable case of arboviral encephalitis is a clinically compatible illness occurring during the season when arbovirus transmission is likely to occur and with the following supportive serology: a stable (twofold or smaller change) elevated antibody titer to an arbovirus, e.g., > 320 by hemagglutination inhibition, > 128 by complement fixation, > 256 by immunofluorescence, > 160 by neutralization, or a positive serologic result by enzyme immunoassay or MAC ELISA.

We recognize that exposure to mosquito-borne disease occurs often and in many locations. Confirming the moment an infected mosquito inoculates a human is nearly impossible to document. Therefore, we assumed human cases were exposed to WNV at their home addresses. The latitude and longitude point locations were provided to the third decimal degree and aggregated to the hexagon level for analytical and display purposes. Human cases were converted into binary form (presence/absence of illness) and weekly case rate, controlling for human population, for each hexagon. Use of human case data was approved by the University of Illinois Institutional Review Board and the Illinois Department of Public Health.

**Abiotic Predictors**

Land Cover: The 2011 United States Geological Survey National Land Cover Database (NLCD) provided 30 m resolution classified raster data for the NWMAD. The raster comprising NWMAD was clipped, extracted, and tabulated by landscape code using the tabulate area tool in ArcGIS 10.5.1. There were 15 unique land cover types: forests (deciduous, evergreen, and mixed), urban (developed open space, developed low intensity, developed medium intensity, and developed high intensity), open water, herbaceous wetlands, cultivated crops, wetlands (woody and herbaceous), grassland, barren land, and shrubs. Proportions of each type within each hexagon were calculated using the 30 m raster resolution.

Weather: Daily mean temperature and precipitation were acquired from the PRISM Climate Group, provided as 4-km resolution grids. Weekly mean temperatures were calculated by taking the average of each of the seven days of the week, whereas weekly precipitation totals were calculated as a sum of each of the seven days of the week. As a proxy for winter temperature, the monthly average for each January from 2005-2016 was also calculated. Using the zonal statistics as table function in ArcGIS, each mean temperature and precipitation value was extracted for each hexagon in this study.

**Biotic Predictors**

Mosquito infection: All mosquito infection data were acquired from the Illinois Department of Public Health (IDPH), the state agency responsible for collecting and maintaining standardized mosquito collection and testing data. Mosquito infection is defined as the minimum infection rate (MIR), calculated by the following equation:

$\frac{\# of positive mosquito pools}{total specimens tested}$ x 1000,

where a mosquito pool in this analysis consisted of up to 50 female *Culex* mosquitoes that were collected by the same trap in the same week. A vast majority of the tests used to identify the presence of WNV was the Rapid Analyte Measurement Platform (RAMP), although some mosquito pools were also tested by Real Time reverse transcriptase polymerase chain reaction (RT-PCR) or VecTest.

Mosquito trap locations were provided by the IDPH. Whenever precise spatial locations were not available, the existing address on file was used to generate a geocoded trap location. The MIR values for each trap were calculated and interpolated across the NWMAD by inverse distance weighting (IDW) in ArcGIS, and resulted in low standard error values, indicating spatial dependency is present (Fig S1). The average MIR values were extracted for each hexagon by using the zonal statistics as table function in ArcGIS.

Demographic: Total population and racial composition (White, African American, Hispanic, and Asian) at the census block level were extracted from the 2010 U.S. Census and was then converted as a percentage for each hexagon. Additionally, age of housing (built before 1940, 1940-1969, 1970-1989, and post 1990) and income were averaged for each hexagon using data provided by 2015 American Community Survey. These data were processed in ArcGIS using the intersection tool.

**Supplemental Results**

**Model Fitting**

With the exception of model E_4_C_3_O_1_, all models successfully converged (Tables S4 and S5), with AUC for the logistic models ranging from 0.84 to 0.97 and BIC values of 576 to 769, while BIC values for linear regression models ranged from -227444 to -181982. Despite converging, all global models (n=8) were excluded from the analysis due to statistical overfitting.
