# Supplementary material for: Dynamics of data availability in disease modeling: An example evaluating the trade-offs of ultra-fine-scale factors applied to human West Nile virus disease models in the Chicago area, USA
Source: PLoS One. 2021 May 19;16(5):e0251517. doi: 10.1371/journal.pone.0251517 (PMC8133451; doi:10.1371/journal.pone.0251517)
Supplement: Supplementary file 8 [file pone.0251517.s002.docx]

| HexID | Cases | Residual | Category | Field Season |
|---|---|---|---|---|
| **4349** | 0 | -1.001 | NR0 | 1 |
| **4806** | 0 | -1.001 | NR0 | 1 |
| **4241** | 0 | -1 | LR0 | 1 |
| **4854** | 0 | -1 | LR0 | 1 |
| **5250** | 0 | -1 | LR0 | 1 |
| **4250** | 1 | -0.271 | LR1 | 1 |
| **4471** | 2 | 0.877 | PR1 | 1 |
| **4183** | 2 | 0.902 | PR1 | 1 |
| **4984** | 1 | 0.912 | PR1 | 1 |
| **5188** | 0 | 1.134 | PR0 | 1 |
| **4597** | 1 | 1.531 | PR1 | 1 |

| HexID | Cases | Residual | Category | Field Season |
|---|---|---|---|---|
| **4014** | 0 | -1.001 | NR0 | Both |
| **4082** | 0 | -1.001 | NR0 | Both |
| **4217** | 0 | -1.001 | NR0 | Both |
| **4415** | 0 | -1.001 | NR0 | Both |
| **4467** | 0 | -1 | LR0 | Both |
| **5199** | 0 | -1 | LR0 | Both |
| **5286** | 0 | -1 | LR0 | Both |
| **4313** | 3 | 0.033 | LR1 | Both |
| **5235** | 1 | 0.055 | LR1 | Both |
| **4609** | 2 | 0.399 | LR1 | Both |
| **4637** | 1 | 1.027 | PR1 | Both |
| **4332** | 1 | 1.279 | PR1 | Both |
| **4335** | 1 | 1.767 | PR1 | Both |
| **4676** | 1 | 1.838 | PR1 | Both |
| **4449** | 3 | 1.841 | PR1 | Both |
| **5239** | 0 | 2.198 | PR0 | Both |
| **4743** | 2 | 4.881 | PR1 | Both |
| **5181** | 1 | 17.057 | PR1 | Both |
| **4617** | 0 | 18.013 | PR0 | Both |

| HexID | Cases | Residual | Category | Field Season |
|---|---|---|---|---|
| 4242 | 0 | -1.001 | NR0 | 2 |
| 4614 | 0 | -1.001 | NR0 | 2 |
| 4181 | 0 | -1 | NR0 | 2 |
| 4381 | 0 | -1 | NR0 | 2 |
| 4382 | 0 | -1 | NR0 | 2 |
| 4578 | 0 | -1 | NR0 | 2 |
| 4923 | 0 | -1 | NR0 | 2 |
| 5185 | 0 | -1 | NR0 | 2 |
| 5262 | 0 | -1 | NR0 | 2 |
| 5234 | 1 | -0.338 | LR1 | 2 |
| 4070 | 1 | -0.32 | LR1 | 2 |
| 4952 | 1 | -0.193 | LR1 | 2 |
| 4073 | 1 | -0.038 | LR1 | 2 |
| 4678 | 1 | -0.014 | LR1 | 2 |
| 4135 | 1 | -0.002 | LR1 | 2 |
| 4104 | 1 | 0 | LR1 | 2 |
| 4334 | 1 | 0.024 | LR1 | 2 |
| 4243 | 2 | 0.071 | LR1 | 2 |
| 5126 | 1 | 0.11 | LR1 | 2 |
| 4065 | 1 | 0.181 | LR1 | 2 |
| 5265 | 1 | 0.221 | LR1 | 2 |
| 4098 | 1 | 5.557 | PR1 | 2 |
| 4636 | 1 | 6.481 | PR1 | 2 |
| 4346 | 1 | 6.967 | PR1 | 2 |
